# Expression of Gibberellin Metabolism Genes and Signalling Components in Dwarf Phenotype of Breadfruit (*Artocarpus altilis*) Plants Growing on Marang (*Artocarpus odoratissimus*) Rootstocks

**DOI:** 10.3390/plants9050634

**Published:** 2020-05-15

**Authors:** Yuchan Zhou, Steven J. R. Underhill

**Affiliations:** 1 Queensland Alliance for Agriculture and Food Innovation, University of Queensland, St Lucia, QLD 4072, Australia; sunderhi@usc.edu.au; 2 Australian Centre for Pacific Islands Research, University of the Sunshine Coast, Sippy Downs, QLD 4556, Australia

**Keywords:** breadfruit (*Artocarpus altilis*), dwarfing, gibberellin (GA), rootstock, stem elongation, marang (*Artocarpus odoratissimus*)

## Abstract

Breadfruit (*Artocarpus altilis*) is a traditional staple tree crop throughout the tropics. The species is an evergreen tree 15–20 m; there are currently no size-controlling rootstocks within the species. Through interspecific grafting, a dwarf phenotype was identified in breadfruit plants growing on Marang (*Artocarpus odoratissimus*) rootstocks, which displayed ~60% reduction in plant height with ~80% shorter internodes. To gain insight into the molecular mechanism underlying rootstock-induced dwarfing, we investigated the involvement of gibberellin (GA) in reduction of stem elongation. Expression of GA metabolism genes was analysed in the period from 18 to 24 months after grafting. In comparison to self-graft and non-graft, scion stems on marang rootstocks displayed decrease in expression of a GA biosynthetic gene, *AaGA20ox3*, and increase in expression of a GA catabolic genes, *AaGA2ox1*, in the tested 6-month period. Increased accumulation of DELLA proteins (GA-signalling repressors) was found in scion stems growing on marang rootstocks, together with an increased expression of a DELLA gene, *AaDELLA1*. Exogenous GA treatment was able to restore the stem elongation rate and the internode length of scions growing on marang rootstocks. The possibility that GA deficiency forms a component of the mechanism underlying rootstock-induced breadfruit dwarfing is discussed.

## 1. Introduction

Trees with reduced stature allow high-density planting and facilitate tree management and harvesting. In many species, tree dwarfing has been achieved through the widespread use of dwarfing rootstocks. The mechanism underlying rootstock-induced dwarfing has been extensively studied but remains poorly understood [1,2,3,4]. Breadfruit (*Artocarpus altilis*) is a traditional staple tree crop throughout the tropics. The species is an evergreen tree from 15 to 20 m. Breadfruits comprise fertile and sterile diploids and sterile triploids and has hundreds of cultivars [5], but there is currently no size-controlling rootstock within the species. Through interspecific grafting, a dwarf phenotype was recently identified in breadfruit plants growing on marang (*Artocarpus odoratissimus*) rootstocks [6]. Under the same genus of *Artocarpus*, marang is also a large tropical fruit tree to 25 m; no dwarf phenotype has been identified [7]. Little is known about the intriguing interaction by which marang greatly reduces the tree size of grafted scions when used as rootstocks.

The molecular mechanism by which dwarfing is conferred may differ between species as well as between annuals and perennials [4]. Several mechanisms have been proposed to explain how rootstocks cause dwarfing in scions. These include reduction of water and solute transport across graft union [3,8], anatomical change [2], and altered hormone signalling between scions and rootstocks [1,9]. There is considerable evidence to suggest that disruption in gibberellin (GA) metabolism plays a role in rootstock-induced dwarfing [4]. Previously dwarfing apple interstocks were found to limit the supply of [^3^H]GA_4_ to scion shoot tips as compared to non-dwarfing interstocks [10]. Dwarfing apple rootstocks also reduce the supply of the root-produced GA precursor, GA_19_, to scions [9,11]. Application of GA to scions on apple dwarfing rootstock restores the node number of both the primary axis and secondary shoots [12]. In ungrafted apple rootstocks, the level of GAs is the lowest in the dwarfing rootstock M.9 and the highest in the non-dwarfing rootstock MM111 [13]. Transcriptomic analysis revealed downregulation of GA biosynthetic genes in scions of apple trees gowning on dwarfing rootstocks [14] and upregulation of a GA catabolic gene together with decreased GA level in persimmon scion stems grafted on dwarfing interstocks [15]. Furthermore, a dwarf plum hybrid with elevated transcript levels of a major GA catabolic gene, *GA2ox*, exhibits shorter internodes and reduced stem elongation, and when used as rootstocks, it reduces the level of bioactive GAs in scions and reduces scion vigor [16].

GAs are a family of diterpenoid plant hormone involved in a wide range of plant growth and development [17]. In higher plants, the flux of active GAs is regulated by the balance between the rates of biosynthesis and deactivation [18]. Biosynthesis of GAs starts from geranylgeranyl diphosphate, a C20 precursor [18]. GA20-oxidase (GA20ox) is a multifunctional enzyme that converts GA_12_ or GA_53_ to GA_9_ or GA_20_ through three sequential oxidations, therefore representing one of the key enzymes controlling GA biosynthetic flux [18]. Suppression of *GA20ox* genes reduces endogenous active GA content and produces dwarfism in many species [17,19,20]. On the other hand, the main route for GA deactivation is through 2β-hydroxylation catalysed by 2-oxyglutarate dependent GA2-oxidases (GA2ox), leading to formation of biologically inactive GAs [17]. Overexpression of *GA2ox* genes therefore enhances GA deactivation and produces dwarf phenotype [16,21]. In parallel with the direct regulation of endogenous GA concentration mediated by GA biosynthetic and catabolic genes, GA signalling is regulated by the negative regulators, DELLA proteins [22]. DELLA proteins are characterized by a highly conserved N-terminal DELLA domain essential for GA-induced proteolysis [23]. Binding of GA molecule to its receptor, GA-INSENSITIVE DWARF1 (GID1) results in rapid degradation of DELLA proteins via the ubiquitin-proteasome pathway, as a result, it releases the DELLA repression of GA responses [23,24]. Mutants with over-accumulated DELLA display dwarf phenotype [23,25]. In plants, GA20-oxidase and GA2-oxidase are encoded by gene families [17], and DELLAs in dicot species are encoded by small gene families of various sizes [23]. While the overlap expression pattern suggests functional redundancy, the tissue-specific expression patterns among family members of GA2-orxidase genes in both poplar [26] and *Arabidopsis* [27] reflect specialised and functional divergence in their relative contribution to GA metabolism and signalling in a subset of organs.

Three predicted functional GA20-oxidases genes were isolated in breadfruit (*Artocarpus altilis* cv. Cannonball), with two genes, *AaGA20ox1* and *AaGA20ox3*, predominantly expressed in green vegetative organs [28]. A cohort of four GA2-oxidase genes, *AaGA2ox1*–*AaGA2ox4*, was also cloned in the same cultivar, with three highly expressed in vegetative organs [29]. There are two DELLA genes, *AaDELLA1* and *AaDELLA2*, isolated in “Mason” breadfruit species [30]. However, the lack of dwarf varieties and the scarcity of dwarfing rootstocks for breadfruits has limited our understanding of how vegetative growth is controlled by rootstocks in the species and any potential role of these genes in conferring breadfruit dwarfism.

Our current work focused on the involvement of GA in the scion stem elongation of breadfruits growing on marang rootstocks in relation to dwarf phenotype. We investigated GA response and the expression of GA biosynthetic and catabolic genes together with DELLA protein abundance and transcript levels in breadfruit scion stems in the period from 18 to 24 months after grafting. Our evidence suggests that GA deficiency may form a component in breadfruit dwarfing mechanism induced by marang rootstocks. The current work provided insight into the molecular mechanism modulating dwarfing in breadfruit through interspecific rootstocks.

## 2. Results

### 2.1. Effect of Rootstocks on Stem Elongation of Breadfruit Scions

**Figure 1:**
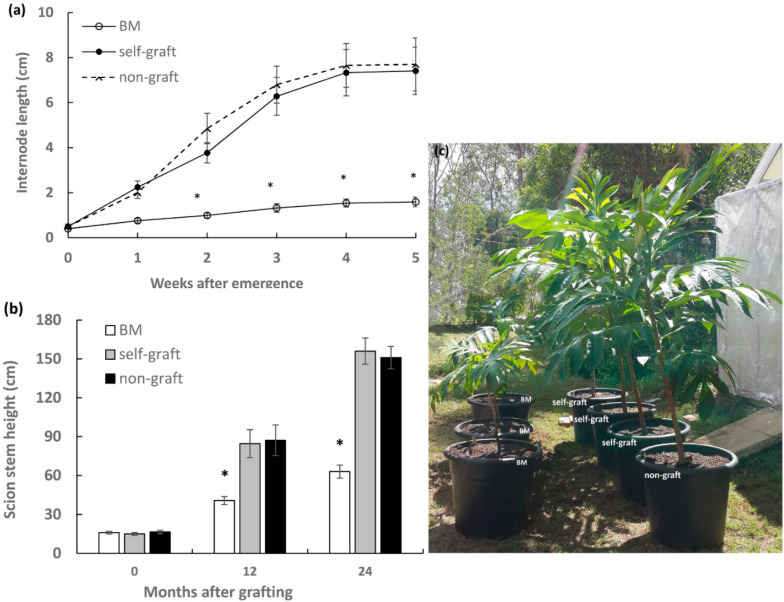
Effect of rootstocks on stem elongation of breadfruit scions: (**a**) Internode elongation in breadfruit scions. Internodes were examined on grafted plants of 20 month olds (after grafting) with measurement initiated from the emergence of the internode under the terminal buds (week 0). (**b**) Height of scion stem growing on different rootstocks. (**c**) Representatives of breadfruit plants growing on different rootstocks at 24 months after grafting. BM, breadfruit plants on marang rootstocks. All values represent mean ± SE from five biological replicates (* *p* < 0.05).

Plants 20 months old after grafting were used for stem elongation analysis. The measurement was started from the emergence of an internode until the cessation of extension in the same segment. Significantly shorter internodes were observed in breadfruit plants growing on marang rootstocks, with 79.8% reduction in final internode length compared to those on self-grafts (Figure 1a). As a result, breadfruit plants on marang rootstocks displayed short stature with height reduction by 52.0% at 12 months and by 59.6% at 24 months after grafting (Figure 1b,c). There was no significant difference in both internode length and final plant height between the self-grafts and the non-graft (Figure 1). The results were consistent with previous growth observation in the 18-month period after grafting [6].

### 2.2. Effect of Rootstocks on the Expression of GA Metabolic Genes

**Figure 2:**
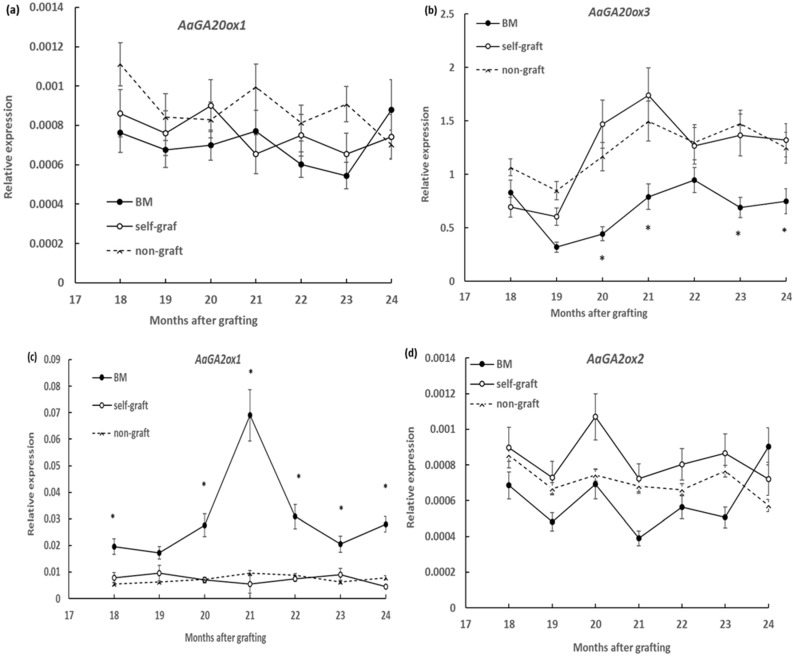
Effect of rootstocks on the expression of gibberellin (GA) biosynthetic genes, *AaGA20ox1* and *AaGA20ox3*, and GA catabolic genes, *AaGA2ox1* and *AaGA2ox2*, in breadfruit scion stems: Expression level of each transcript was normalized to the expression of the actin gene. BM, breadfruit plants growing on marang rootstocks. All values represent mean ± SE from five separate RNA extractions (* *p* < 0.05). (**a**) *AaGA20ox1*; (**b**) *AaGA20ox3*; (**c**) *AaGA2ox1* and (**d**) *AaGA2ox2*.

Six GA20-oxidase genes, *AaGA20ox1*–*AaGA20ox6*, were previously isolated in breadfruits, with three, *AaGA20ox1*–*AaGA20ox3*, predicted to encode functional GA20-oxidase and the other three, *AaGA20ox4*–*AaGA20ox6*, predicted to be unprocessed pseudogenes [28]. The expression of the three *AaGA20oxs*, *AaGA20ox1–AaGA20ox3*, were measured in scion stems, but only two genes, *AaGA20ox1* and *AaGA20ox3*, showed expression in the current experiment condition. Similarly, of the four *GA2ox* genes previously identified in breadfruit [29], only two genes, *AaGA2ox1* and *AaGA2ox2*, were detected in scion stem tissues under the current growth condition. All the detectable genes were analysed monthly in the period from 18 to 24 months after grafting. It was shown that the overall expression levels of *AaGA20ox3* were over 1000 times higher than those of *AaGA20ox1* and that the expression levels of *AaGA2ox1* were over 10 times higher than those of *AaGA2ox2* for all samples (Figure 2a–c). Over the six month period, scion stems on marang rootstocks showed no significant change in transcript levels of *AaGA20ox1* but showed decreased transcript levels of *AaGA20ox1* at serval time points, including 20, 21, 23, and 24 months after grafting when compared to those on self-grafts and non-grafts (Figure 2a,b). There was no significance difference between the self-graft and non-graft in the expression of these two genes (Figure 2a,b). For GA2-oxidase genes, higher levels of expression were detected every month from 18 to 24 months except for the 19-month time point for *AaGA2ox1*, but no significant change for *AaGA2ox2* compared to those on self-grafts and non-graft was observed (Figure 2c,d). For both *AaGA2ox1 and AaGA2ox2,* the expression levels of the self-graft and non-graft were not significantly different (Figure 2c,d).

### 2.3. Effect of Rootstocks on DELLA Protein Abundance and Transcript Levels

Two DELLA genes, *AaDELLA1* and *AaDELLA2*, were previously cloned from “Mason” breadfruit [30]. To confirm that the two genes or potentially more DELLA genes were present in stems of “Gold Noli” breadfruit, DELLA genes were first cloned from “Gold Noli” breadfruit by degenerate PCR using primers corresponding to two conserved regions of all known DELLAs, MDELLA(V/A) and AHFTANQA [23]. An expected fragment of 700 bp was amplified, and 40 degenerate PCR clones were sequenced. Only two distinct groups of cDNA clones were identified with one aligned to *AaDELLA1* and the other aligned to *AaDELLA2*. Both alignments had 100% similarity to the previous *AaDELLAs*. Full lengths of the two DELLA genes were then isolated using primers corresponding to the 5’ and 3’ end of the *AaDELLA1* and *AaDELLA2* genes. Sequencing of the resulting full-length cDNA clones confirmed that “Mason” breadfruit had two DELLA genes, one identical to *AaDELLA1* and the other identical to *AaDELLA2* at the nucleic acid level.

**Figure 3:**
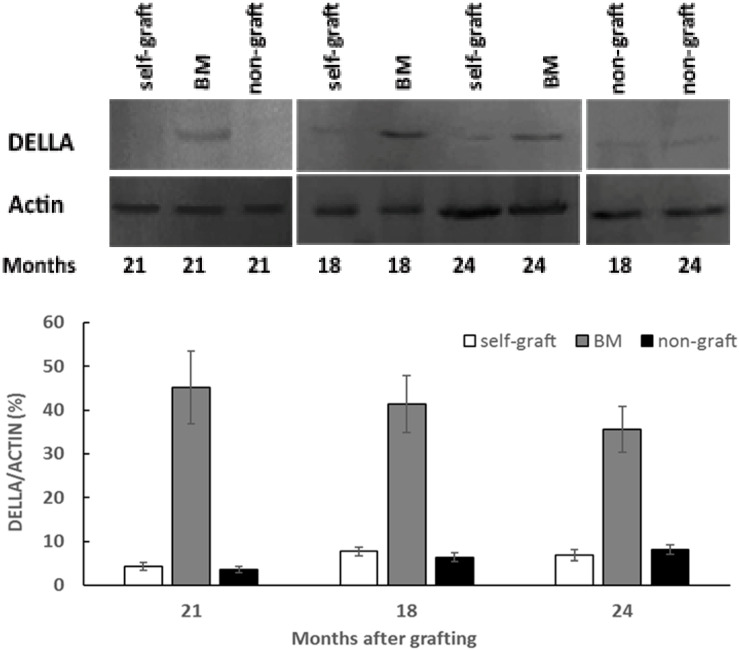
Analyses of DELLA protein abundance in breadfruit scion stems on different rootstocks: Total proteins were extracted from scion stems at 18, 21, and 24 months after grafting. DELLA protein abundance was determined by Western blotting with affinity-purified polyclonal anti-AaDELLA. A representative immunoblot of DELLA protein with actin as a loading control is shown on top of the histogram showing the ratio of the band intensity of the DELLA to that of the corresponding actin. BM, breadfruit plants grafted on marang rootstocks. Vertical bars represent mean ± SE derived from three biological replicates.

**Figure 4:**
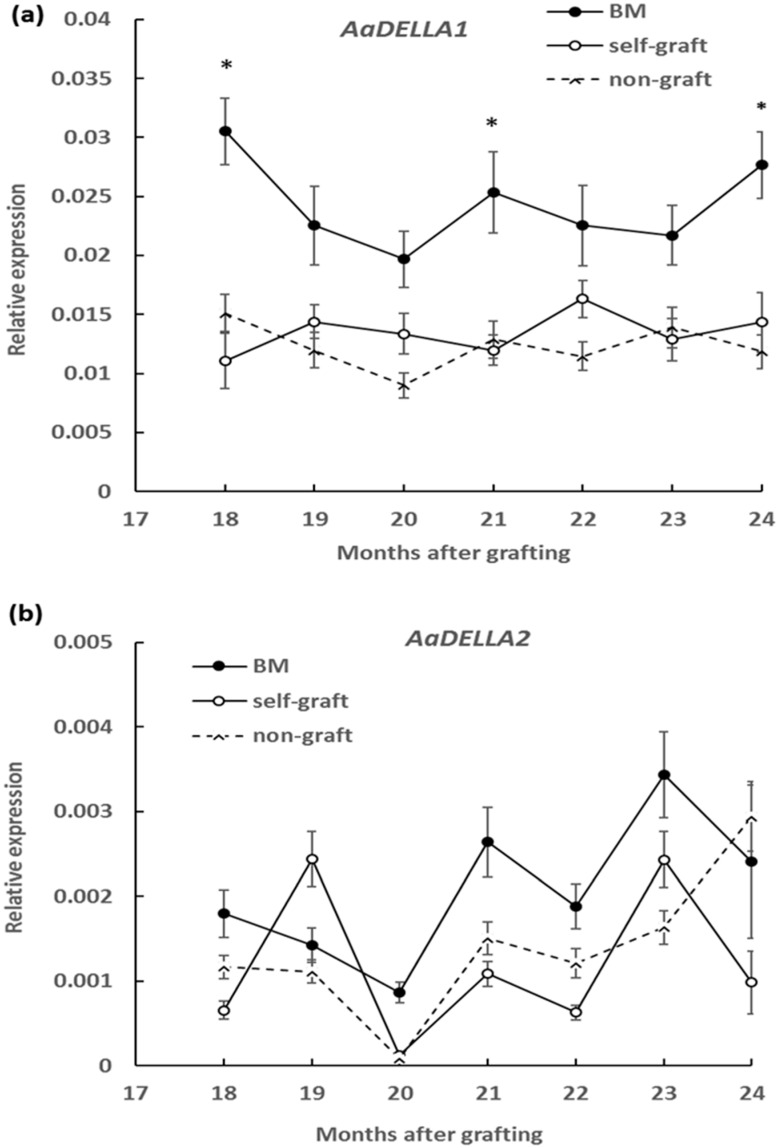
Effect of rootstocks on the expression of DELLA genes, *AaDELLA1* and *AaDELLA2*, in breadfruit scion stems: Expression level of each transcript was normalized to the expression level of the actin gene. BM, breadfruit plants growing on marang rootstocks. All values represent mean ± SE from five separate RNA extractions (* *p* < 0.05). (**a**) *AaDELLA1*; (**b**) *AaDELLA2.*

To measure the DELLA protein abundance, a polyclonal anti-AaDELLA was raised which targeted the highly conserved region of AaDELLA1 and AaDELLA2 (see Materials and Methods). DELLA protein abundance was examined in scion stems growing on different rootstocks every 3 months in the period of 18 to 24 months after grafting. Compared to the self-graft and non-graft at the corresponding times, levels of immunologically detectable DELLA were obviously higher in scion stems grafted on marang rootstocks, indicating an increase in the accumulation of DELLA proteins at all the tested time points (Figure 3). The transcript levels of *AaDELLA1* and *AaDELLA2* were further analysed in scion stems growing on different rootstocks. When compared to those on the self-graft and non-graft over the 6-month period, scion stems on marang rootstocks were found to have increases in the expression of *AaDELLA1* at three time points, including 18, 21, and 24 months, but to have relatively stable expression of *AaDELLA2* (Figure 4). The expression levels between the self-graft and the non-graft were not significantly different for both genes (Figure 4).

### 2.4. Restoration of Stem Elongation by GA Treatment

**Figure 5:**
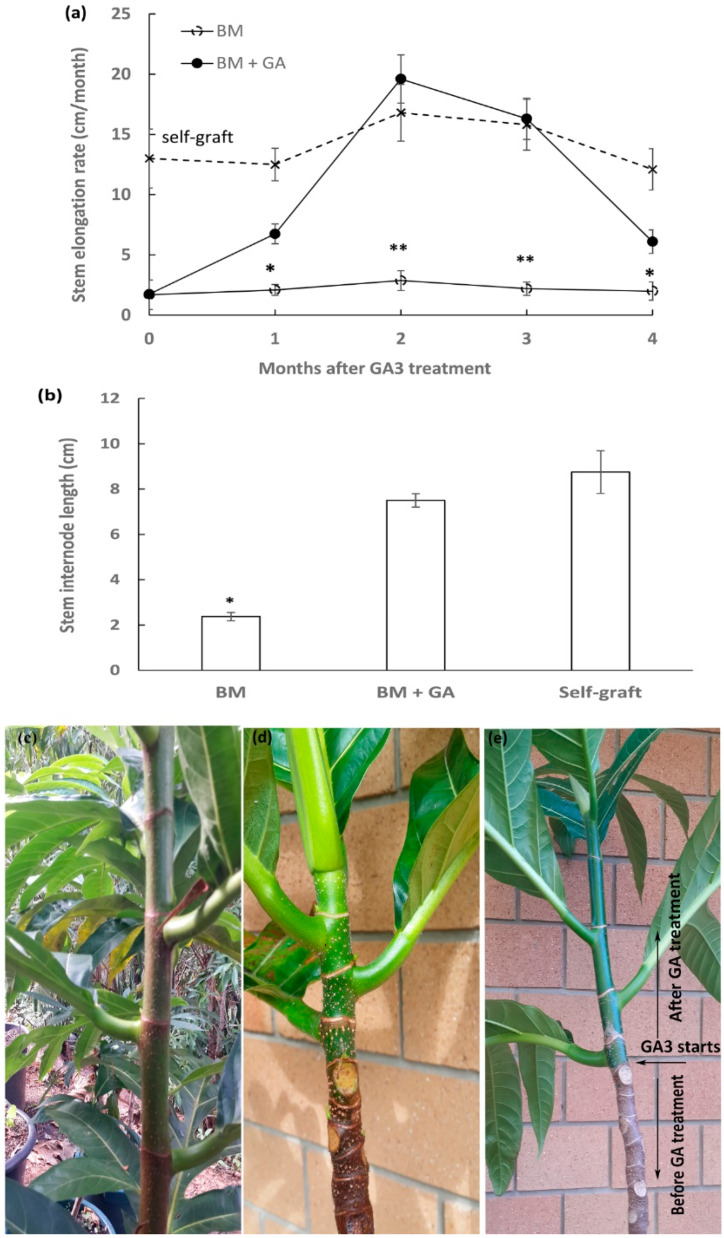
Rescue of scion stem elongation by exogenous GA_3_ application in breadfruit plants on marang rootstocks: Comparison of stem elongation rate (**a**) and internode length (**b**) in GA_3_-treated and non-treated plants. Representatives of grafted plants displaying different scion stem internodes: (**c**) Untreated breadfruit scions on breadfruit rootstock (self-graft), (**d**) untreated breadfruit scions on marang rootstock, and (**e**) GA_3_-treated breadfruit scions on marang rootstock. The stem internode length was the averaged measurement of the second internodes at the third month after GA_3_ treatment. BM, breadfruit plants growing on marang rootstocks. All values represent mean ± SE from three biological replicates, with ** significant difference (*p* < 0.05) from the rest of the samples (non-graft and self-graft) and * significant difference from the non-graft only.

To confirm that the rootstock-induced short stature is due to GA deficiency, breadfruit plants growing on marang rootstocks were sprayed with GA_3_. For GA treatment, the plants were 18 months old (after grafting) with a single stem, characterised by the main active stem elongation from the terminal buds and minimal growth at the lower part of the stems. Following GA application, it was shown that their stem elongation rate was significantly increased, with 7.5-fold higher in the second month and 6.4-fold higher in the third month compared to those of the untreated plants on marang rootstocks at the same time (Figure 5a). By the 3rd month after exogenous GA application, the stem elongation rate was fully restored to normal with no significant difference to those on self-graft (Figure 5a). Furthermore, the internode length was increased by 2.2-fold after 3 months following GA treatment (Figure 5) and was also restored to nearly normal compared to those on self-graft (Figure 5b). There was no significant change in the number of internodes and the stem thickness within the four months after GA_3_ treatment.

## 3. Discussion

Breadfruit plants growing on marang rootstocks were characterised by dwarf stature with ~60% reduction in total plant height and ~80% reduction in the length of internodes at the end of 24 months after grafting (Figure 1), consistent with the previous observation in the first 18-month period [6]. The phenotypes resemble the typical signs of GA deficiency [21,31]. The expression of GA biosynthetic genes and catabolic genes in scion stems growing on different rootstocks was compared over the period from 18 to 24 months after grafting. Of the two *GA20o*x genes expressed in stems, the predominant gene, *AaGA20ox3*, showed significantly reduced levels of expression several times during the six-month period (Figure 2). These suggest that the capacity of GA biosynthesis in scions of marang rootstocks may be affected by the reduced expression of the *AaGA20ox3* gene during the period. Our results are in agreement with another study, where downregulation of the GA biosynthetic genes was found in scions gowning on dwarfing apple rootstocks of both M.9 and M.27 [14]. Suppression of *GA20ox* expression has been shown to produce plants with dwarf stature as a result of reduced endogenous GA contents in many species, inducing *Arabidopsis*, potato, tobacco, [17], apple trees [19], and citrus trees [20]. These suggest that downregulation of *AaGA20ox3* may contribute to the dwarf phenotype of breadfruit plants grafted on marang rootstocks. Of the two GA catabolic genes, *AaGA2ox1* and *AaGA2ox2*, a more consistent increase in the transcript levels of the major gene, *AaGA2ox1*, in the period suggests an upregulation of GA deactivation in scion stems on marang rootstocks in this period (Figure 2). Upregulation of a GA2ox gene accompanying a decreased GA level was previously reported in scions of persimmon trees grafted on dwarfing interstocks [15]. Overexpression of *GA2ox* enhances GA deactivation and produces dwarf phenotype in many species [16,17,21]. These results suggest that upregulation of the main GA catabolic gene, *AaGA2ox1*, may contribute to the rootstock-induced dwarf phenotype in breadfruit plants over the period from 18 to 24 months after grafting.

The combination of upregulation of a GA biosynthetic gene and downregulation of a GA catabolic gene suggests a decrease in GA signals in scion stems growing on marang rootstocks. In support of this hypothesis, an increase in the accumulation of DELLA proteins (GA-signalling repressors) was found in scion stems on marang rootstocks (Figure 3). Various evidences have previously indicated that dwarfing rootstocks reduce GA concentration in scions [9,10,11]; the change of DELLA protein abundance has rarely been reported. This work provides insight into the functional significance of the GA deficiency in relation to the repression of GA response. In plants, DELLA proteins are rapidly degraded in response to GA via the ubiquitin proteasome pathway [23,24]; an increased DELLA protein abundance therefore reflects an increased repression of GA response in scions grafted on marang rootstocks. DELLA proteins act as negative regulators of plant growth [23]. Plants carrying over-accumulated DELLA display dwarf phenotype [25,32,33]. DELLAs also interact with multiple transcription factors and key regulators of other pathways through direct protein–protein interactions [22]. These include integrating GA with brassinosteroid, ethylene, jasmonate, abscisic acid, and auxin signalling pathways [22,34,35,36]. Some of these signal pathways have long been proposed to be implicated in rootstock-induced dwarfism of other species [4]. In this context, the role of GA and its interaction with other growth regulators in regulating the dwarf traits of breadfruit on marang rootstocks deserves further investigation. Collectively, our results suggest that an increased repression of GA response contribute to the dwarf phenotype in breadfruit plants growing on marang rootstocks. These may be due to reduced GA signals as a result of the downregulation of GA biosynthesis and/or upregulation of GA deactivation. On the other hand, the increase in DELLA protein levels was reflected by the increased transcript levels of a DELLA gene, *AaDELLA1*, at all three occasions (Figure 4), suggesting that *AaDELLA1* may be regulated at the transcription level. While most previous work has demonstrated the posttranscriptional regulation of DELLA protein [22,23], the transcriptional regulation of DELLA in response to various environmental signals has also been reported [35,37]. Upregulation of a *DELLA* gene was found in scions growing on persimmon dwarfing interstocks. In this context, it may be possible that transcriptional regulation of the *AaDELLA1* plays a coordinative role in regulating GA response, thus contributing to the mechanism of rootstock-induced dwarfism in breadfruit plants.

Stem elongation of scions on marang rootstocks was restored to near normal by exogenous GA application as determined by both the stem elongation rate and stem internode length (Figure 5). The results provide evidence that GA deficiency may play a role in rootstock-induced dwarfing of breadfruit plants and support the association of scion GA metabolism genes and signalling genes, including *AaGA20ox3*, *AaGA2ox1*, and *AaDELLA1*, with the development of dwarf phenotype in breadfruit plants. The combined expression profiles of these genes therefore may represent potential markers for breadfruit dwarfing. Breadfruit tree height can be controlled though the use of the GA inhibitor paclobutrazol [29]. For woody species, this generally involves a long-term, repeated application of the chemicals in order to achieve effective tree-size reduction. Our current work may provide an opportunity to develop various size-controlling rootstocks through fast screening of GA-related gene markers in scions, leading to environmentally sustainable solutions for breadfruit dwarfing.

In the current study, we focused on stem elongation as a key phenotype in breadfruit plants on marang rootstocks. However, the rootstock-induced dwarf traits in breadfruits involve other morphological and biochemical components [6] which are not examined in the current experiment. Future characterisation of the roles of GA and other hormones/factors in these dwarf traits is required for the unravelling of the molecular mechanism underlying rootstock-induced breadfruit dwarfing. GA is also produced in roots [18], although the majority of the studies have suggested that GA precursors rather than active GA are involved in long-distance transport [38]. Previously, dwarfing apple rootstocks were shown to limit the root-produced GA precursor, GA_19_, to scions [9,10,11]. While our study suggests a reduction of GA response in scion stems as determined by the increase of DELLA protein abundance, the nature of root-derived GAs and their contribution to the GA response in scion stems need further investigation. On the other hand, phloem transport of DELLA mRNA from rootstocks to scions has been reported [39]. The possibility of marang DELLA gene transfer to breadfruit scions cannot be ruled out. The sequence of marang *DELLA* gene is not available, but it is possible that the polyclonal anti-DELLA in the current study also binds marang-derived DELLAs, given that the target region shares high homology to DELLA sequences of other species (NCBI BLAST search).

In conclusion, breadfruit scion stems growing on marang rootstocks displayed decreased expression of a major GA biosynthetic gene, *AaGA20ox3*, at several times over the 6-month period from 18 to 24 months after grafting, and had persistently higher expression of a major GA catabolic gene, *AaGA2ox1*. Increased DELLA protein abundance was shown in scion stems on marang rootstocks together with an increase in the expression of a DELLA gene, *AaDELLA1*. Exogenous GA treatment was able to restore the stem elongation rate and the internode length of scions growing on the dwarfing rootstocks.

## 4. Materials and Methods

### 4.1. Plant Materials and Treatments

Breadfruit (*Artocarpus altilis* cv. Cannonball, also called “Noli Gold”) and marang (*Artocarpus odoratissimus*) plants were obtained from a commercial nursery at Cairns, northern Queensland. Breadfruit plants as rooted cuttings and marang plants as seedlings were grown under glasshouse condition at 25 to 28 °C with natural daylight and daily water supply. Plants were grown in pots containing vermiculite and soil mixture as described previously [29]. Breadfruit scions selected from breadfruit plants 30 to 50 cm tall were grafted onto marang seedlings of similar sizes through approach grafting [40]. As a control, the breadfruit scions were also grafted onto breadfruit rootstocks of the same cultivar (self-graft). There were at least eight replicates for each grafting combination. Each established grafted plant was transferred to an 85-Litre pot six months after grafting and continued to grow under the same condition. The self-rooted breadfruit plants (non-graft) were grown alongside under the same condition. Plants were monitored for total height and stem elongation within the 24 months after grafting. Plants on marang rootstocks 18 months after grafting were used for GA treatment. For GA experiment, both foliage and soil surface were sprayed with 500 mg L^−1^ GA_3_ (Sigma, Sydney, NSW, Australia, dissolved in 0.1% ethanol, 0.1% Triton X-100). The bioactive form GA_3_ was chosen according to the previous experiment, showing a fast positive response to the chemical in stem growth of breadfruit trees [29]. Plants sprayed with the same concentration of ethanol and Triton X-100 were used for control. The treatment was applied once a week for 3 weeks. Three replicates were used for each treatment and control. Plants were monitored for stem elongation, and internode length in the second internode was counted from the top, with the first internode being defined as the one below the uppermost leaf.

### 4.2. Quantitative Real-Time PCR

Upon removal from plants, stem tissues (including epidermis, vascular tissues, and pith) in the second internodes were immediately immersed in RNA*later* (Life Technologies, VIC, Australia) before stored at −80 °C. Total RNA was extracted by using RNeasy kit (Qiagen, VIC, Australia) and reverse transcribed with SuperScript reverse transcriptase and oligo(dT) (Life Technologies, VIC, Australia). Real-time PCR was performed on a Corbett Research Rotor-Gene 6000 cycler with the QuantiFast SYBR Green PCR Kit (Qiagen, VIC, Australia) as previously described [29]. Thermocycling was initiated with a 5-min incubation at 95 °C, followed by 40 cycles (95 °C for 10 s; 60 °C for 30 s). The specificity of amplification was confirmed by high-resolution melt curve analysis at the end of each run. The efficiency of each primer set was evaluated by standard curves using serial dilutions of plasmid DNA containing its amplified regions. Each reaction was carried out in duplicate (technical repeat) with non-reverse-transcribed cDNA (RT^−^) as negative controls (non-template control). Transcripts of *AaGA20oxs* and *AaGA2oxs* were amplified using their gene-specific primers as previously described [28,29]. Gene-specific primers were 5′-GAA AAA GATCAGAAGAAGAAGAATCATCATG-3′ and 5′-CCC AAA ACG GCC AGG AGCTCG-3′ for *AaDELLA2 for AaDELLA1* and 5′-CAT CAG AAG AAGAAT CAT GAA AAG GGA AC-3′ and 5′-CAT GTC GGATGA CCT GAC CTT GTA G-3′ for *AaDELLA2*. Two housekeeping genes, actin and elongation factor 1-α (*AaEFα-1*) were tested for stability across the time points according to a previous protocol [41]. The actin gene was amplified using primers 5’-AATGGAACTGGAATGGTGAAG GC-3’ and 5’-TGCCAGATCTTCTCCATGTCATCC-3’, and the *AaEFα*-1 was amplified using primer 5’-GAAGCTCTTCGTCAAGAGAA-3’ and 5’-GAAATCTCTTGAAGTAACCATC-3’. The specificity of the primers was confirmed by amplicon sequencing. The actin gene, a more stable housekeeping gene compared to the elongation factor 1-α gene, was chosen to normalize to the expression of transcript abundance. The expression of each gene was an average of five biological replicates.

### 4.3. Cloning of DELLA cDNAs from “Gold Noli” Breadfruit

Total RNA was extracted from stem tissues of breadfruit plants by using RNeasy kit RNeasy kit (Qiagen, VIC, Australia) and reverse transcribed with reverse transcriptase and oligo(dT) (Life Technologies, VIC, Australia). The resulting cDNA was subjected to degenerate PCR using primers 5′-ATGGAYGARYTIYTIGCNG-3′ and 5′-GCNCAYTTYACNGCNAAYCARGCN-3′ as previously described [30]. The PCR reactions were performed at 35 cycles with annealing temperature at 52 °C. The PCR products were cloned into pGEMT vector (Promega, NSW, Australia) and sequenced. Full-length cDNA clones of *AaDELLA* genes were amplified with Advantage DNA polymerase (Takara Clontech, CA, USA). The thermocycling was initiated with a 2-min incubation at 92 °C, followed by 32 cycles (92 °C for 30 s and 68 °C for 2 min) and a final extension of 7 min at 68 °C. The gene-specific primers were 5′-GAAAAAGATCAGAAGAAGAAGAATCATCATG-3′ and 5′-GACCCGACTTAGCGAGCCACAG-3′ for *AaDELLA1* and were 5′-GTGTTTGAGGAAAAAGAGGGCCTGTG-3′ and 5′-GGGTCCGGCCCGACTCAGAG-3′ for *AaDELLA2.* These primers were designed from the sequence information of *AaDELLA* genes isolated from the “Mason” breadfruit [30]. PCR products were cloned into pGEMT vector and sequenced as above. The resulting sequences were analysed by Sequencher (version 5.4.1, Gene Codes Corporation, Ann Arbor, MI, USA).

### 4.4. Western Blotting

The highly conserved sequence of AaDELLA1 and AaDELLA2 [30] between residues 37 and 59 (KMWEEDDGGMDELLAVLGYKVR) was synthesized, and the resulting peptide was used to raise antibodies in a rabbit (Mimotopes, Melbourne, Australia). Polyclonal anti-AaDELLA was purified by affinity chromatography. The titres of the affinity-purified antibodies were determined by enzyme-linked immunosorbent assay (ELISA). Proteins were extracted from the full stem tissues in the second internodes of scions according to the method previously described [42], and protein concentration was determined by a bicinchoninic acid protein assay (Sydney, NSW, Australia). The protein extracts were resolved on 10% SDS-PAGE gels (20 µg protein per lane) and electroblotted onto nitrocellulose membranes followed by immuno-detection [43]. Blots were probed with pre-immune serum as negative controls. Colour development was performed using an alkaline phosphatase-conjugated secondary antibody with Western blue (Promega, NSW, Australia). Each blot was first probed with polyclonal anti-AaDELLA; then stripped with 0.2 N glycine, pH 2.5; and re-probed with anti-actin (Sydney, NSW, Australia). The ratio of the band intensity of the DELLA to that of the corresponding actin was analysed by Quantity One 1-D analysis software in the Gel Doc system (BioRad, NSW, Australia).

### 4.5. Statistical Analyses

Significant differences were tested using analysis of variance (ANOVA) followed by Tukey’s multiple comparison test at *p* < 0.05 (IBM SPSS Statistics version 24).
